# Oxygen and contact with human intestinal epithelium independently stimulate virulence gene expression in enteroaggregative Escherichia coli

**DOI:** 10.1111/cmi.13012

**Published:** 2019-02-15

**Authors:** Samuel J. Ellis, Muhammad Yasir, Douglas F. Browning, Stephen J.W. Busby, Stephanie Schüller

**Affiliations:** ^1^ Norwich Medical School University of East Anglia Norwich UK; ^2^ Quadram Institute Bioscience Norwich UK; ^3^ Institute of Microbiology and Infection University of Birmingham Birmingham UK

**Keywords:** adherence, colonic epithelium, diarrhoea, EAEC, gene expression, oxygen, virulence

## Abstract

Enteroaggregative Escherichia coli (EAEC) are important intestinal pathogens causing acute and persistent diarrhoeal illness worldwide. Although many putative EAEC virulence factors have been identified, their association with pathogenesis remains unclear. As environmental cues can modulate bacterial virulence, we investigated the effect of oxygen and human intestinal epithelium on EAEC virulence gene expression to determine the involvement of respective gene products in intestinal colonisation and pathogenesis. Using in vitro organ culture of human intestinal biopsies, we established the colonic epithelium as the major colonisation site of EAEC strains 042 and 17‐2. We subsequently optimised a vertical diffusion chamber system with polarised T84 colon carcinoma cells for EAEC infection and showed that oxygen induced expression of the global regulator AggR, aggregative adherence fimbriae, E. coli common pilus, EAST‐1 toxin, and dispersin in EAEC strain 042 but not in 17‐2. Furthermore, the presence of T84 epithelia stimulated additional expression of the mucinase Pic and the toxins HlyE and Pet. This induction was dependent on physical host cell contact and did not require AggR. Overall, these findings suggest that EAEC virulence in the human gut is modulated by environmental signals including oxygen and the intestinal epithelium.

## 1 INTRODUCTION

Enteroaggregative Escherichia coli (EAEC) are emerging foodborne pathogens of worldwide importance. First described in 1987, they have been associated with persistent infantile diarrhoea in the developing world (Nataro et al., 1987). More recent studies indicate that EAEC are also a common cause of traveller's diarrhoea, important enteric pathogens in HIV‐patients, and associated with large foodborne outbreaks in industrialised countries (Estrada‐Garcia & Navarro‐Garcia, 2012; Hebbelstrup Jensen, Olsen, Struve, Krogfelt, & Petersen, 2014). Notably, a large prospective study on infectious intestinal disease has reported EAEC infection as the second most common cause of bacterial diarrhoea in the United Kingdom (after *Campylobacter*; Tompkins et al., 1999). In addition, “hypervirulent” Shiga toxin‐producing EAEC strains are emerging, causing potentially fatal systemic disease that cannot be treated with antibiotics. The severity of this has been underlined by a large EAEC outbreak in Germany in 2011 that resulted in 1,000 hospitalisations and 50 deaths (Bielaszewska et al., 2011).

Despite their considerable impact on human health, the mechanisms of how EAEC cause disease remain unknown. This is partly due to a lack of suitable animal models that reflects their specificity for the human host (Philipson, Bassaganya‐Riera, & Hontecillas, 2013). In addition, EAEC are a heterogenous group, and not all strains cause human disease (Jenkins, Chart, Willshaw, Cheasty, & Tompkins, 2007; Nataro et al., 1995). The differences in pathogenicity among EAEC isolates can probably be attributed to their traditional classification based on “stacked brick”‐like aggregative adherence to HEp‐2 cells, which does not necessarily reflect their ability to cause human disease (Nataro et al., 1987). Nevertheless, research on EAEC so far indicates that bacterial adherence to intestinal epithelium, biofilm formation, release of toxins, and mucosal inflammation likely contribute to pathogenesis and diarrhoea (Estrada‐Garcia & Navarro‐Garcia, 2012).

During the last two decades, several putative virulence factors including adhesins, serine protease autotransporters (SPATEs), and toxins have been described (Estrada‐Garcia & Navarro‐Garcia, 2012). Typical EAEC strains possess an aggregative adherence plasmid (pAA), which encodes the AraC‐like DNA‐binding protein AggR (Nataro, Yikang, Yingkang, & Walker, 1994). AggR acts as a transcriptional activator for a multitude of genes including those encoding aggregative adherence fimbriae (AAF; Morin, Santiago, Ernst, Guillot, & Nataro, 2013; Nataro et al., 1994). AAF are a class of fimbrial adhesins strongly associated with aggregative adherence, and five alleles (AAF/I‐V) have been described so far (Jønsson et al., 2015). Protein interaction studies have identified several AAF host receptors including cytokeratin 8, MUC1, and extracellular matrix proteins (Boll et al., 2017; Izquierdo et al., 2014). Similar to AAF, dispersin is plasmid‐encoded and regulated by AggR and has been linked to adherence and biofilm formation (Sheikh et al., 2002). Structural studies suggest that dispersin binding to outer membrane lipopolysaccharide masks its negative charge and allows positively charged adhesins such as AAF to bind more distant sites, thereby promoting dispersal of adherent bacteria (Velarde et al., 2007). Although AAF are strongly linked to aggregative adherence, many EAEC isolates have no AAF allele, and the phenotype is believed to be multifactorial (Jønsson et al., 2015). For example, the E. coli common pilus (ECP) found in many E. coli pathotypes (Rendón et al., 2007) has been implicated in aggregative adherence, especially in AAF‐negative strains (Avelino et al., 2010). In addition to adhesins, EAEC produce several toxins, which are important for the induction of diarrhoeal symptoms. The enterotoxin EAST‐1 has similarities to the heat‐stable enterotoxin STa of enterotoxigenic E. coli and is proposed to function in a comparable way via interference of cGMP signalling and dysregulation of anion secretion (Ménard, Lussier, Lepine, Paiva de Sousa, & Dubreuil, 2004). Haemolysin E (HlyE) is a pore‐forming toxin mediating cytolytic and cytopathic effects in cultured human cells. As it is also found in nonpathogenic bacteria, the role of HlyE in EAEC pathogenesis remains unclear (Navarro‐Garcia & Elias, 2011). Another enterotoxin associated with EAEC virulence is the plasmid‐encoded SPATE Pet that degrades the structural protein spectrin, leading to cytoskeletal disruption in epithelial cells (Boisen, Ruiz‐Perez, Scheutz, Krogfelt, & Nataro, 2009; Canizalez‐Roman & Navarro‐García, 2003). In addition, some EAEC strains express the SPATE Pic that cleaves mucins and complement proteins and stimulates mucus hypersecretion in the gut (Henderson, Czeczulin, Eslava, Noriega, & Nataro, 1999; Navarro‐Garcia et al., 2010).

Despite the identification of these and other putative virulence factors, genotypic studies have failed to consistently associate a single gene or combination of genes with EAEC pathogenicity (Estrada‐Garcia & Navarro‐Garcia, 2012). This may be due to the heterogeneity of the EAEC pathotype and/or differences in regulation of virulence gene expression. Previous studies, particularly on enterohaemorrhagic E. coli (EHEC), have demonstrated that the intestinal environment influences bacterial virulence gene expression and thereby promotes selection of the optimal niche for bacterial survival and colonisation of the host (Barnett Foster, 2013; Carlson‐Banning & Sperandio, 2017). Here, we have investigated the effect of oxygen levels and human colonic epithelium on EAEC virulence gene expression by employing a microaerobic (MA) vertical diffusion chamber (VDC) with polarised human intestinal epithelial cells (Schüller & Phillips, 2010).

## 2 RESULTS

### 2.1 EAEC strains 17‐2 and 042 adhere to human colonic but not small intestinal epithelium

**Figure 1:**
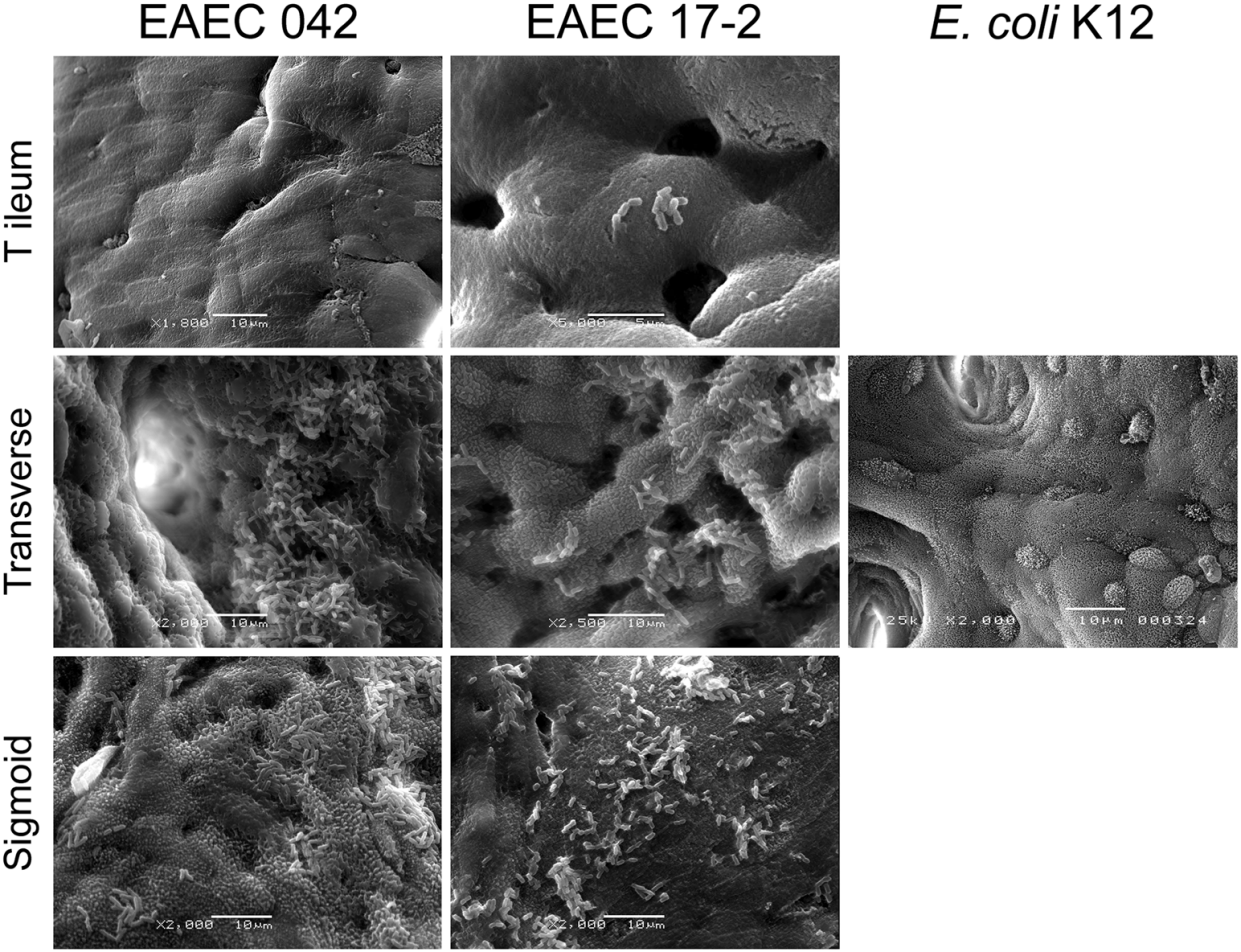
Scanning electron microscopy of human biopsies from the terminal ileum, transverse, and sigmoid colon infected with enteroaggregative *Escherichia coli* (EAEC) strains 042 or 17‐2 or Escherichia coli K12 (negative control) for 7 hr. Bars = 10 μm except EAEC 17‐2 T ileum where bar = 5 μm. Shown are representative images of three experiments performed in duplicate

**Figure 2:**
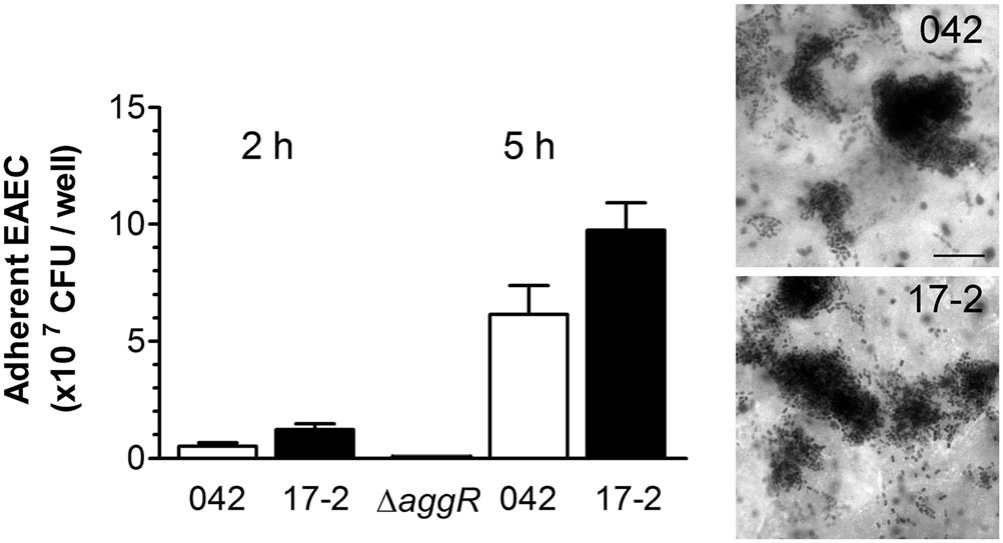
Adherence of EAEC 042 and 17‐2 to T84 cells. Colonisation was quantified after 2 and 5 hr of infection and expressed as colony forming units (CFU) per well; 042 Δ*aggR* was included as negative control (*n* = 3 in duplicate). Aggregative adherence after 5 hr was visualised by Giemsa staining. Bar = 5 μm

For this study, we employed the well‐characterized EAEC prototype strains 17‐2 and 042, which have been used in human volunteer studies (Nataro et al., 1995). To select the most suitable intestinal epithelial cell line for the VDC infection model, we first evaluated EAEC adherence in in vitro organ culture (IVOC) of human mucosal biopsies from different parts of the intestine (proximal small intestine to distal colon). Although both EAEC strains demonstrated aggregative adherence to tissue from the transverse and sigmoid colon after 7 hr of incubation, only few bacteria bound to biopsies from the terminal ileum (Figure 1), and no bacteria were detected on duodenal samples (Table 1). Aggregative adherence to colonic mucosa was specific for EAEC as no epithelium‐bound bacteria were detected on biopsy samples incubated with E. coli K12 (Figure 1). Aggregative adherence and colonisation of colonic epithelium was confirmed by infecting human colon carcinoma‐derived T84 cells with EAEC for up to 5 hr (Figure 2), and this cell line was subsequently used for further studies.

**Table 1:** Adherence of enteroaggregative *Escherichia coli* to human intestinal biopsies

Region	EAEC strain	
	042	17‐2
Duodenum	0/8 (0)	0/8 (0)
Terminal ileum	4/7 (57)^a^	3/7 (43)^a^
Transverse colon	6/7 (86)	4/7 (57)
Sigmoid colon	6/7 (86)	2/6 (33)

*Note*. Data are presented as number of biopsies with adherent aggregates per total number of biopsies (percentage).

a Single bacteria or small aggregates only.

### 2.2 Establishment of the MA VDC system

**Figure 3:**
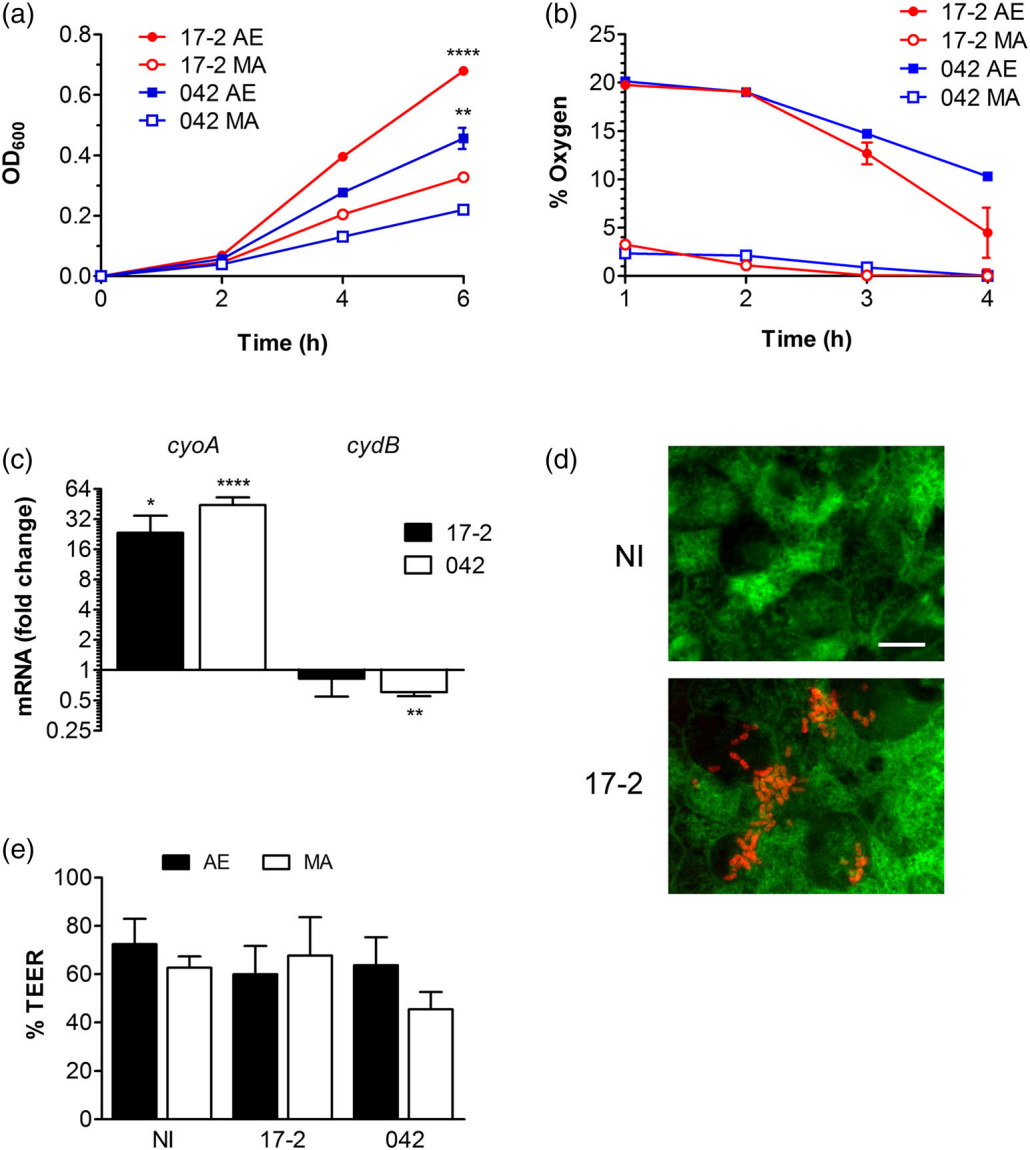
Optimisation of the vertical diffusion chamber system for EAEC infection. (a–c) Chambers without T84 cells were inoculated with EAEC strains and incubated under aerobic (AE) or microaerobic (MA) conditions for various time periods. (a) Bacterial growth was quantified by optical density (OD_600_); ^**^ *P* < 0.01, ^****^ *P* < 0.0001 versus MA conditions. (b) Oxygen concentrations were determined as percentage of atmospheric pressure. (c) Bacterial respiration status after 3 hr of incubation was evaluated by qPCR analysis of low affinity cytochrome *bo*_*3*_ oxidase (*cyoA*) and high affinity cytochrome *bd* oxidase (*cydB*). Gene expression is indicated as fold change under AE versus MA conditions; ^*^ *P* < 0.05, ^**^ *P* < 0.01, ^****^ *P* < 0.0001 (*n* = 3 in duplicate). (d,e) Chambers with polarised T84 cells were infected with EAEC or left non‐infected (NI) for 3 hr under MA or AE conditions. (d) Aggregative adherence and epithelial integrity was visualised by immunofluorescence staining for EAEC (red) and actin (green). Bar = 5 μm. Shown are representative images of four experiments. (e) Epithelial barrier function was evaluated by transepithelial electrical resistance (TEER) and is expressed as resistance after infection relative to resistance before infection (*n* = 3 in duplicate)

In our previous studies, we have shown that T84 cells grown on Snapwell supports form polarised and well‐differentiated epithelial monolayers that can be maintained in the MA VDC for at least 6 hr without loss of barrier function or cell viability (Schüller & Phillips, 2010; Tran, Billoud, Lewis, Phillips, & Schüller, 2014). To optimise the system for EAEC infection, we first determined the growth kinetics of strains 17‐2 and 042 under aerobic (AE) and MA conditions and simultaneously quantified the concentrations of dissolved oxygen in the media. Although both strains demonstrated growth in the VDC system, this was significantly higher under AE versus MA conditions (Figure 3a). Measurement of dissolved oxygen levels showed AE (20%) and MA (2–3%) oxygen concentrations at the beginning of the incubation with subsequent oxygen depletion during bacterial growth (Figure 3b). Oxygen consumption was more pronounced in chambers inoculated with strain 17‐2, which correlated with faster growth of this strain compared with 042. As oxygen concentrations in AE chambers incubated with 17‐2 reached near MA levels (5%) after 4 hr, a period of 3 hr was chosen for subsequent bacterial gene expression analysis. Differences in bacterial respiration status between AE and MA conditions were confirmed by analysis of terminal oxidase expression. In E. coli, AE respiration in oxygen‐rich environments is mediated by the low affinity cytochrome *bo*_*3*_ oxidase complex (*cyoABCDE*), whereas the high affinity cytochrome *bd* oxidase (*cydAB*) is utilised at low‐oxygen tensions (Cotter, Chepuri, Gennis, & Gunsalus, 1990; Morris & Schmidt, 2013). As shown in Figure 3c, both EAEC strains expressed significantly increased levels of *cyoA* and reduced levels of *cydB* under AE versus MA conditions, thereby confirming that oxygen concentrations in AE chambers were still sufficiently high to enable AE respiration via cytochrome *bo*_*3*_ oxidase. In addition to bacterial growth and respiration, we evaluated EAEC host cell interactions in the VDC system. After 3 hr of infection, both strains showed aggregative adherence to polarised T84 cells under AE and MA conditions without significantly affecting monolayer integrity or transepithelial electrical resistance (Figure 3d, image shown for 17‐2 only, and Figure 3e).

### 2.3 Oxygen induces virulence gene expression in EAEC 042

**Figure 4:**
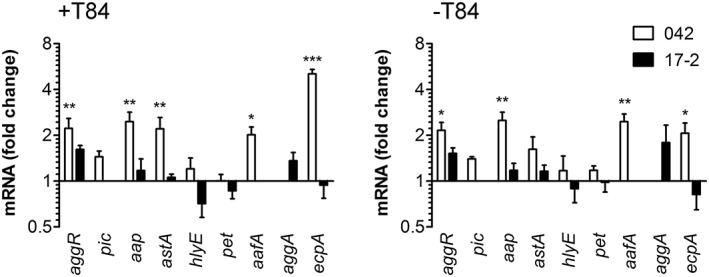
Oxygen enhances virulence gene expression in EAEC 042. Strains 17‐2 or 042 were incubated in the vertical diffusion chamber with (+) or without (−) polarised T84 cells for 3 hr under aerobic (AE) or microaerobic (MA) conditions. Expression of selected virulence genes in planktonic bacteria was determined by qPCR and is indicated as fold change under AE versus MA conditions (*n* = 3 in duplicate for +T84, *n* = 5 in duplicate for −T84). ^*^ *P* < 0.05, ^**^ *P* < 0.01, ^***^ *P* < 0.001

To determine the influence of oxygen concentrations on bacterial virulence gene expression, polarised T84 cells were infected with strains 17‐2 or 042 for 3 hr under AE or MA conditions. After harvesting nonadherent bacteria from the apical media, RNA was extracted and transcription of selected virulence genes was determined by qPCR (Table 2). Whereas little effect was observed in strain 17‐2, expression of *aggR*, *aap*, *astA*, *aafA*, and *ecpA* was significantly enhanced under AE versus MA conditions in strain 042 (Figure 4). To evaluate if any of these effects were mediated by changes in the host cells, incubations were performed in chambers without T84 cells. Similar results were obtained except for *astA* and *ecpA* where lower induction levels were observed for strain 042 (Figure 4).

**Table 2:** EAEC virulence factors examined in this study

Type	Gene	Protein	Present in
Regulator	*aggR*	AggR	042, 17‐2
Adhesins	*aafA*	AAF/II structural subunit	042
	*aggA*	AAF/I structural subunit	17‐2
	*ecpA*	ECP structural subunit	042, 17‐2
Toxins	*astA*	EAST‐1	042, 17‐2
	*hlyE*	HlyE	042, 17‐2
	*pet*	Pet	042, 17‐2
Other	*pic*	Pic	042
	*aap*	Dispersin	042, 17‐2

*Note*. EAEC: enteroaggregative Escherichia coli.

### 2.4 Host cell contact enhances EAEC virulence gene expression

**Figure 5:**
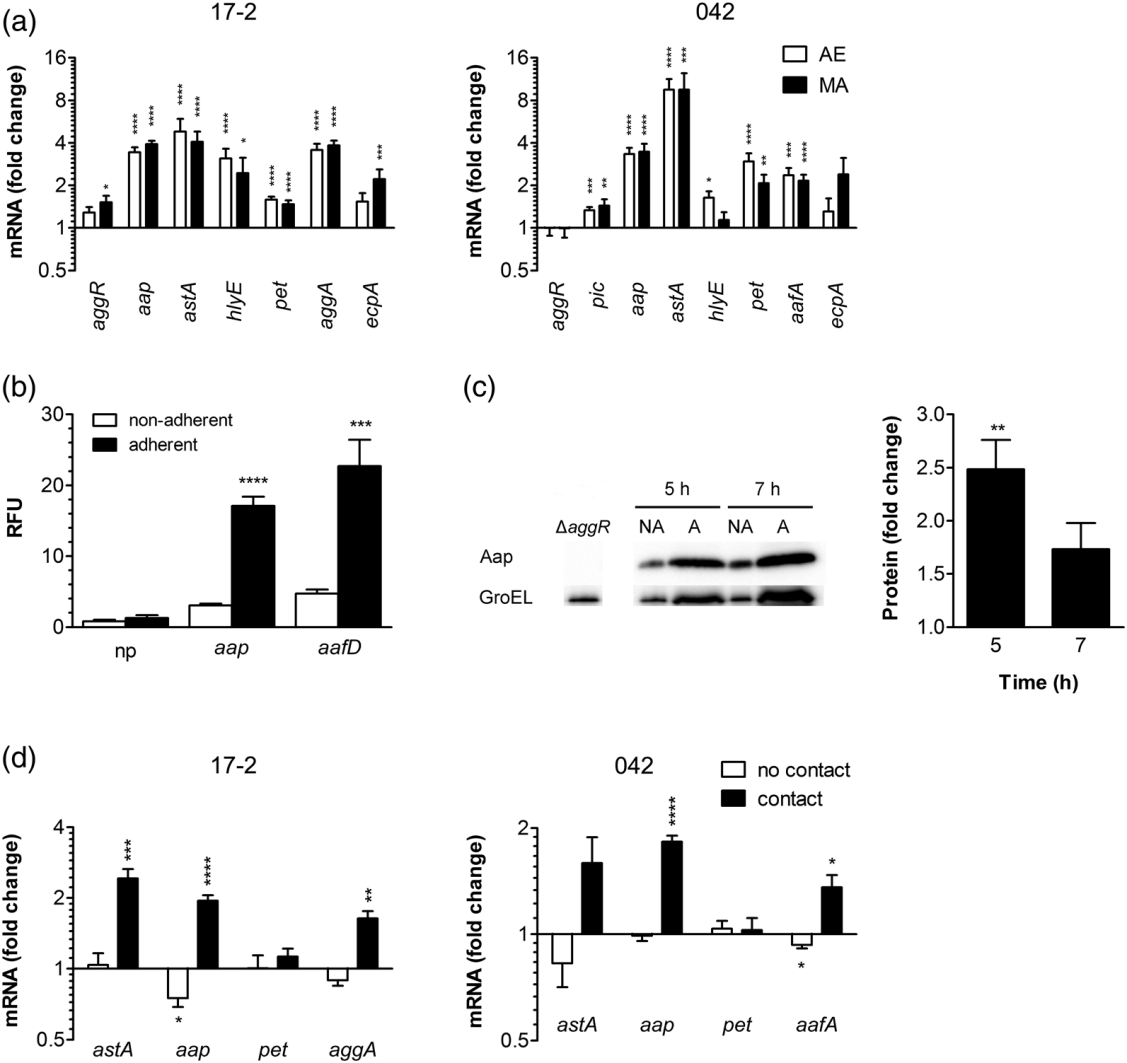
EAEC virulence gene expression is enhanced by host cell contact. (a) Polarised T84 cells were infected with EAEC 17‐2 or 042 and maintained under aerobic (AE) or microaerobic (MA) conditions for 3 hr. Expression of selected virulence genes in cell‐bound and planktonic EAEC in the medium was determined by qPCR and is indicated as fold change in adherent versus nonadherent bacteria (*n* = 5 in duplicate). (b) Confluent T84 cells grown in 24 well plates were infected with EAEC DFB042TC carrying promoterless *gfp* (np), *aap*500‐*gfp* (*aap*), or *aafD*100‐*gfp* (*aafD*) reporter constructs for 5 hr. GFP expression in adherent and nonadherent bacteria was determined by fluorescence intensity and normalised to colony forming units (CFU). Fluorescence is displayed as relative fluorescence units (RFU) per 10^4^ CFU (*n* = 3 in triplicate). (c) Polarised T84 cells were infected with strain 042 for 5 or 7 hr under AE conditions. Expression of dispersin (Aap) in adherent (A) and nonadherent bacteria (NA) was determined by Western blot analysis. Bacterial lysates of an isogenic *aggR* mutant (Δ*aggR*) were included as negative control. Expression of GroEL was used to normalise total protein amounts. Band intensities were quantified with ImageJ, and protein expression is indicated as fold change in adherent versus nonadherent bacteria (*n* = 3). (d) T84 cells were grown in 12 well plates, and EAEC were added directly to the cells or prevented from direct cell contact by insertion of a porous Transwell insert. After 3 hr, expression of selected virulence genes was quantified by qPCR and is expressed as fold change in EAEC in Transwell inserts with and without T84 cells (no contact) or in adherent versus nonadherent EAEC in plates without Transwells (contact; *n* = 3 in duplicate). ^*^ *P* < 0.05, ^**^ *P* < 0.01, ^***^ *P* < 0.001, ^****^ *P* < 0.0001

We next characterized the influence of host cells on virulence gene expression. Polarised T84 intestinal epithelia were infected in the VDC as described above. After 3 hr, nonadherent (planktonic) and adherent bacteria were harvested for RNA extraction, and gene transcription was analysed by qPCR. In strain 042, all selected genes except *aggR* and *ecpA* were significantly induced in adherent versus planktonic bacteria (Figure 5a). This was comparable under MA and AE conditions apart from *hlyE* that only showed significant upregulation under AE conditions. Similarly, adherent EAEC 17‐2 demonstrated a significant increase in expression of all tested virulence genes compared with nonadherent bacteria (Figure 5a). This was comparable under AE and MA conditions except for *aggR* and *ecpA*, which were significantly induced under MA conditions only. To confirm enhanced virulence gene expression in adherent bacteria, reporter plasmids were generated by fusing the promoters of EAEC 042 *aafD* (*aafA* is transcribed from the upstream *aafD* promoter) or *aap* to *gfp* by using the GFP expression plasmid pRW400. Constructs were subsequently transformed into the tetracycline‐sensitive 042 derivative DFB042TC. Infections of confluent T84 cells were carried out for 5 and 7 hr to allow for GFP expression, and fluorescence of adherent and nonadherent EAEC was determined. Whereas GFP expression in promoterless controls was unaffected, fluorescence of adherent versus nonadherent bacteria was significantly enhanced in reporter strains carrying the *aafD* or *aap* promoter, and this was most pronounced at 5 hr post infection (Figure 5b, data shown for 5 hr infection only). Furthermore, increased dispersin (Aap) expression in adherent versus nonadherent EAEC 042 was confirmed by Western blot analysis of bacterial lysates that reached significance after 5 hr of infection (Figure 5c).

To determine if physical contact between bacteria and host cells was required for virulence gene induction, T84 cells were seeded out in 12 well plates. EAEC were either added directly to the cells or prevented from direct cell contact by addition to a Transwell insert with a porous membrane enabling exchange of soluble mediators. For comparison, bacteria were incubated in Transwell supports in 12 well plates without T84 cells. After bacterial RNA extraction, qPCR was performed for a subset of virulence genes, and gene expression was compared between (a) EAEC in Transwells with and without T84 cells (no bacteria–host cell contact) and (b) adherent and nonadherent EAEC in well plates without Transwells (bacteria–host cell contact). As shown in Figure 5d, expression of all virulence genes except *pet* was increased in adherent versus nonadherent bacteria, which paralleled our findings in the VDC system. In contrast, no induction in gene expression was observed when bacteria were separated from the T84 epithelium by a Transwell insert, in which case, even a significant reduction in expression of *aafA* (042) and *aap* (17‐2) was detected (Figure 5d).

### 2.5 Dependence of virulence gene induction on AggR regulation

**Figure 6:**
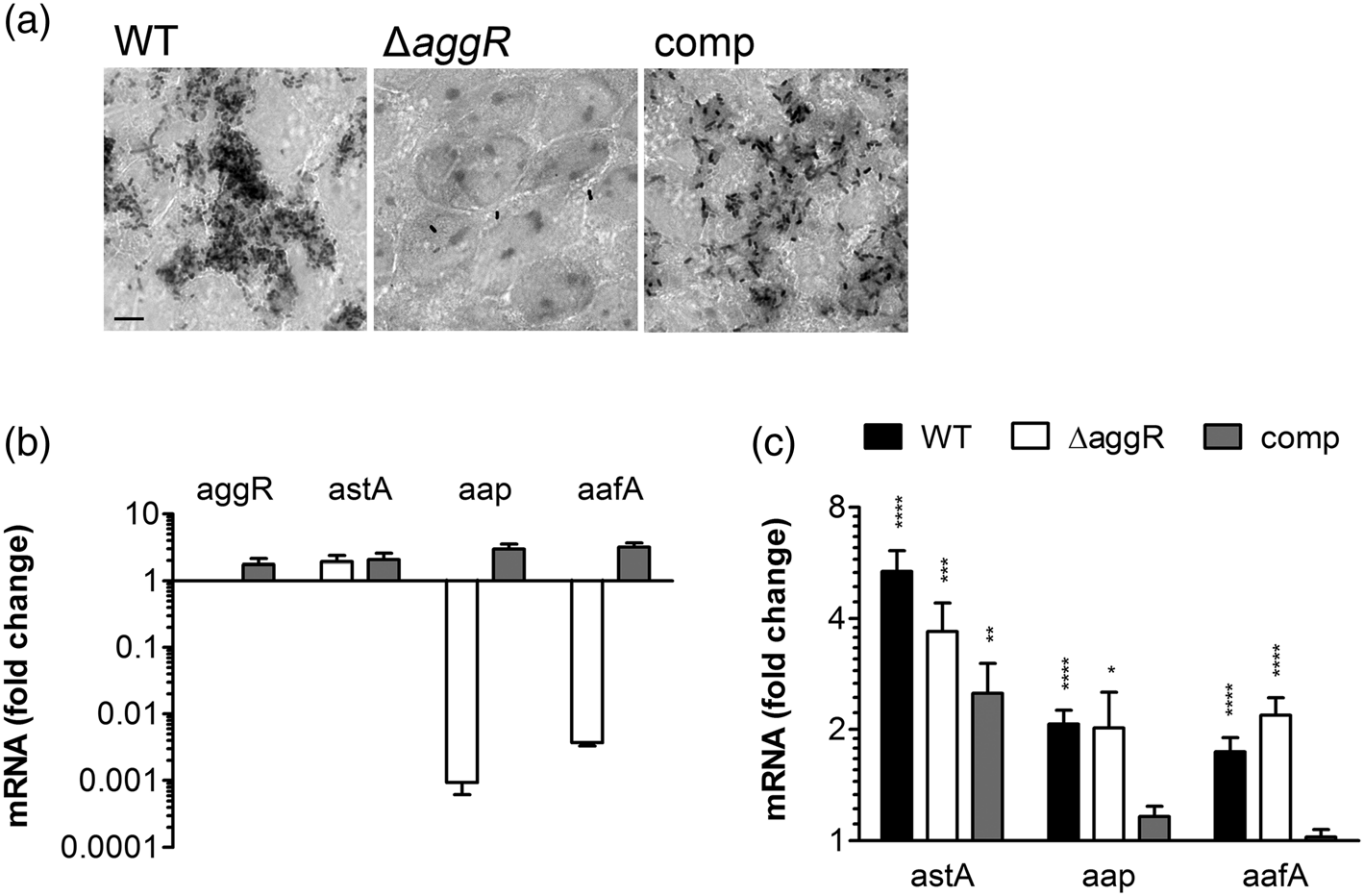
Influence of AggR on aggregative adherence and EAEC virulence gene expression. T84 cells in 24 well plates were infected with EAEC 042 wild type (WT), Δ*aggR* mutant, and complemented strain (comp) for 4 hr. (a) Giemsa stain of infected cells. Bar = 5 μm. Shown are representative images of two experiments performed in duplicate. (b) Virulence gene expression in planktonic bacteria was quantified by qPCR and is expressed as fold change in Δ*aggR* mutant (white) and complemented strain (grey) versus WT (*n* = 4 in triplicate). (c) Host cell‐induced virulence gene expression is not dependent on AggR. Transcription of selected virulence genes in adherent and nonadherent EAEC was determined by qPCR and is indicated as fold change in adherent versus nonadherent bacteria (*n* = 4 in triplicate). ^*^ *P* < 0.05, ^**^ *P* < 0.01, ^***^ *P* < 0.001, ^****^ *P* < 0.0001

To determine the dependency of oxygen‐ and contact‐induced virulence gene expression on the global activator AggR, experiments were conducted using an isogenic 042 *aggR* deletion mutant and plasmid‐complemented strain. Functionality of the mutant strains was validated by infection of confluent T84 cells and evaluation of adherence by Giemsa stain. Whereas 042 wild‐type formed adherent aggregates, the *aggR* deletion mutant showed binding of isolated single bacteria only (Figure 6a). Aggregative adherence was restored in the *aggR*‐complemented strain although bacterial clusters appeared less dense compared with the wild type. In addition, relative gene expression levels of *aggR*, *astA*, *aap*, and *aafA* were determined in nonadherent EAEC harvested from incubations with T84 cells under AE conditions. As shown in Figure 6b, no *aggR* expression and strongly reduced levels of *aap* or *aafA* mRNA were detected in 042 Δ*aggR*, whereas transcription of all three genes was about twofold to threefold higher in the complemented than the wild‐type strain. In contrast, *astA* expression was not affected by AggR and about twofold higher in Δ*aggR* and complemented strain when compared with the wild type. Similar transcription patterns were obtained in adherent EAEC from the same experiments (data not shown). When we investigated the influence of AggR on oxygen‐stimulated virulence gene expression, we did not obtain any conclusive results due to high transcript level variations in the *aggR* deletion mutant and complemented strain (data not shown). However, qPCR analysis of infected T84 cells demonstrated significantly increased *astA*, *aap*, and *aafA* transcript levels in adherent versus nonadherent bacteria for both wild‐type and Δ*aggR* mutant (Figure 6c). Unexpectedly, host cell adherence only stimulated expression of *astA* in the complemented strain whereas transcript levels of *aap* and *aafA* remained unchanged.

## 3 DISCUSSION

Although EAEC are a heterogenous group of E. coli strains defined by aggregative adherence to HEp‐2 cells, we have focused our studies on two well‐characterized prototype strains, 042 and 17‐2, which have been isolated from children with diarrhoeal illness in South America (Nataro et al., 1985; Vial et al., 1988). Both strains are typical EAEC encoding the *aggR* regulon of putative virulence genes commonly associated with pathogenic EAEC strains (Cerna, Nataro, & Estrada‐Garcia, 2003; Dudley, Thomson, Parkhill, Morin, & Nataro, 2006). When tested in adult volunteers, strain 042 induced diarrhoea whereas 17‐2 did not cause any disease (Nataro et al., 1995). Previous studies using human mucosal biopsies demonstrated that both strains preferentially adhered to colonic compared with small intestinal tissue (Hicks, Candy, & Phillips, 1996; Knutton et al., 1992; Nataro, Hicks, Phillips, Vial, & Sears, 1996), which agrees with our IVOC results. T84 colon carcinoma cells were chosen for the establishment of the VDC system as they exhibit structural similarity to intestinal crypt cells and form highly polarised columnar epithelia (Madara, Stafford, Dharmsathaphorn, & Carlson, 1987). In addition, previous studies have shown pAA‐dependent aggregative adherence of EAEC 042 to T84 but not to Caco‐2 cells (Nataro et al., 1996). In contrast, strain 17‐2 forms aggregates on Caco‐2 (Couto, Oliveira, Queiroz, & Freitas‐Almeida, 2007; Steiner, Lima, Nataro, & Guerrant, 1998) and T84 cells as demonstrated in our study.

Although conventional cell culture studies are performed under AE conditions, the gastrointestinal tract is an oxygen‐limited environment with decreasing oxygen levels towards the colon as oxygen is consumed by the resident microbiota. Non‐invasive measurements in living mice and rats demonstrated oxygen levels of 1.4% in the mid‐colon and 2–4% at the small intestinal epithelium, respectively (He et al., 1999; Zeitouni, Chotikatum, Köckritz‐Blickwede, & Naim, 2016), which is comparable to the oxygen concentrations in the MA VDC chamber. As a facultative anaerobe, EAEC can grow under AE and MA conditions. In the VDC system, we observed increased multiplication of both EAEC strains under AE versus MA conditions, which agrees with enhanced expression of the low‐affinity cytochrome *bo*_*3*_ oxidase resulting in a higher energy yield compared with cytochrome *bd* oxidase‐mediated respiration (Gunsalus & Park, 1994; Jones et al., 2007). Although the 3 hr infection period used in our protocol was sufficient for aggregative adherence of both strains, it did not allow EAEC‐mediated disruption of epithelial barrier function (Strauman, Harper, Harrington, Boll, & Nataro, 2010) and subsequent leak of oxygen across the epithelium.

Oxygen availability has been recognised as an important environmental signal for the modulation of virulence in enteropathogenic bacteria (Marteyn, Scorza, Sansonetti, & Tang, 2011). *Salmonella typhimurium* demonstrates increased host cell adherence and invasion when grown under low‐oxygen tension (Lee & Falkow, 1990). In addition, the FNR transcriptional regulator involved in sensing low‐oxygen levels is required for full *Salmonella* virulence in mice and modifies expression of a type III secretion system (T3SS) required for pathogenesis (Fink et al., 2007). Similarly, oxygen modulates T3SS expression and subsequent adherence of EHEC to intestinal epithelium (Schüller & Phillips, 2010). In *Shigella flexneri*, lack of oxygen in the gut lumen enhances expression of the T3SS but suppresses secretion of virulence proteins resulting in their accumulation inside bacteria. As *S. flexneri* approaches the mucosal surface, oxygen released by the epithelium triggers targeted T3S and subsequent bacterial invasion (Marteyn et al., 2010).

Here, we demonstrate an oxygen‐dependent induction of genes associated with adherence (*aafA*, *aap*, and *ecpA*), which might prime the bacteria for host cell binding as they approach the intestinal epithelium. The corresponding upregulation of *aggR*, *aap*, and *aafA* suggests that modulation of the *aggR* regulon is linked to oxygen sensing. This effect is independent on the presence of T84 cells and therefore not mediated by hypoxia‐related changes in epithelial cell function, which have been shown to influence infection by enteropathogenic bacteria (Zeitouni et al., 2016). Notably, induction of adherence gene expression was only evident in strain 042 but not 17‐2. This might be related to strain‐specific differences in gene regulation as an earlier study showed different temperature‐dependent AAF expression in strains 042 and 17‐2 (Hinthong et al., 2015).

In contrast, adhesion to host cells enhanced expression of not only colonisation‐associated virulence genes but also those encoding toxins and SPATEs (*astA*, *hlyE*, *pet*, and *pic*). Stimulation of gene expression in adherent EAEC was largely independent of oxygen levels and evident in both strains, although the extent of induction of specific genes differed between 042 and 17‐2. Regulation of virulence by sensing chemical cues has been widely reported in EHEC (Barnett Foster, 2013), and previous studies on EAEC have shown enhanced *pet* expression in nutrient‐rich versus minimal media (Betancourt‐Sanchez & Navarro‐Garcia, 2009). In our study, however, physical contact with the epithelium rather than host‐secreted soluble compounds were responsible for gene induction in adherent EAEC. Notably, this was more pronounced in the VDC system compared with similar experiments performed in cell culture plates. Although similar inocula and incubation periods were used in both experimental models, the levels and/or kinetics of bacterial gene expression may have been affected by the less polarised state of the T84 cells and enhanced bacteria–host cell contact due to gravity in the culture plate. Recent studies on EHEC have shown that mechanosensation plays an important role in regulation of virulence with initial bacterial surface attachment resulting in induced T3S, which was further exacerbated by fluid shear force (Alsharif et al., 2015). So far, several host cell receptors for AAF‐mediated binding of EAEC have been identified including extracellular matrix proteins, cytokeratin 8, and MUC1 (Boll et al., 2017; Farfan, Inman, & Nataro, 2008; Izquierdo et al., 2014). Although extracellular matrix proteins only become accessible for EAEC binding after epithelial damage, and cytokeratin 8 is not expressed in the cell membrane of normal intestinal epithelial cells (Gires, Andratschke, Schmitt, Mack, & Schaffrik, 2005), MUC1 might represent a target for initial EAEC binding and contact‐dependent induction of virulence gene expression (McGuckin, Lindén, Sutton, & Florin, 2011). Interestingly, AggR was not involved in this response, as an *aggR* deletion mutant still demonstrated adhesion‐induced transcription of *astA*, *aap*, and *aafA*. Previous microarray studies on EAEC grown in Dulbecco's modified Eagle's medium (DMEM) have shown that AggR positively regulates the expression of at least 44 genes including *aap* and *aafA* but not *astA* (Morin et al., 2013). This is confirmed by our results where expression of *aap* and *aafA* was considerably reduced in EAEC Δ*aggR* versus wild‐type strain whereas transcription of *astA* remained largely unaffected in planktonic bacteria grown in DMEM/F‐12 medium. In addition to DMEM, AggR has been shown to enhance *aggA* expression in EAEC 17‐2 under various temperatures, osmolarities, and oxygen tensions. Interestingly, *aggA* expression at acid pH was regulated by both AggR‐dependent and AggR‐independent mechanisms (Nataro et al., 1994) suggesting that certain environmental triggers do not signal via AggR. Alternative regulators likely include the global transcriptional factors CRP and Fis that activate *pet* expression (Rossiter et al., 2011). Unexpectedly, the *aggR*‐complemented strain failed to demonstrate host cell‐induced transcription of *aap* and *aafA*, which might be due to enhanced copy numbers of plasmid‐encoded *aggR* and subsequent dysregulation of gene expression. Notably, this did not affect *astA* expression that is not regulated by AggR.

Overall, our findings suggest that oxygen and host cell contact act as separate signals for EAEC niche adaptation in the human gut. The oxygen gradient encountered as EAEC approach the mucosal surface may prime the bacteria for adherence, whereas contact with the epithelium induces a wider range of virulence factors important for colony expansion and later stages of pathogenesis.

## 4 EXPERIMENTAL PROCEDURES

### 4.1 Bacterial strains and culture conditions

EAEC prototype strains 042 (serotype O44:H18, isolated from a case of paediatric diarrhoea in Peru in 1983; Nataro et al., 1985) and 17‐2 (serotype O3:H2, isolated from a case of infant diarrhoea in Chile, 1988; Vial et al., 1988) were provided by Marie Anne Chattaway (Gastrointestinal Bacteria Reference Unit, Public Health England). Mutant strains 042 Δ*aggR* and 042 *aggR*::pBAD30 have been described previously (Sheikh et al., 2002). Strain DFB042TC was constructed by sequentially disrupting the *cat* and *tetA* genes in EAEC 042, which encode for chloramphenicol and tetracycline resistance, respectively. The *cat* gene was disrupted by introducing an internal stop codon into *cat*, using the suicide plasmid pCVD442 (Donnenberg & Kaper, 1991). The *tetA* gene was disrupted using gene doctoring methodology (Lee et al., 2009) in which *tetA* was replaced by a kanamycin resistance cassette and then removed. The construction of GFP reporter strains is described below. E. coli strain K12 was purchased from New England Biolabs. For infections, bacteria were grown standing in lysogeny broth (LB‐Lennox, Formedium) overnight at 37°C. For selection of mutants, 50 μg/ml kanamycin, 100 μg/ml ampicillin, or 15 μg/ml tetracycline were added as appropriate. Expression of *aggR* by 042 *aggR*::pBAD30 was induced with 0.2% (*w*/*v*) L‐arabinose.

### 4.2 IVOC and scanning electron microscopy

This study was performed with approval from the University of East Anglia Faculty of Medicine and Health Ethics Committee (ref 2010/11‐030). All samples were provided through the Norwich Biorepository, which has NRES approval (ref 08/h0304/85+5). Biopsy samples from the second part of the duodenum, terminal ileum, transverse colon, and sigmoid colon were obtained from consenting adult patients undergoing routine endoscopy at the Gastroenterology Department of the Norfolk and Norwich University Hospital. Samples were taken from areas without any macroscopic inflammation or abnormality. IVOC was performed as described previously (Lewis, Cook, Tighe, & Schüller, 2015). Briefly, biopsies were mounted on a foam support with the mucosal side facing upwards and inoculated with 25 μl EAEC overnight culture (~2.5 × 10^7^ colony forming units [CFU]) or left non‐infected. Tissue samples were incubated on a rocking platform at 37°C in a 5% CO_2_ atmosphere. After 7 hr of incubation, biopsies were washed in PBS to remove mucus and nonadherent bacteria, fixed in 2.5% glutaraldehyde in PBS, and dehydrated through graded acetone series. Specimens were dried using tetramethylsilane (Sigma), sputter‐coated with gold (Polaron SC7640, Quorum Technologies), and imaged with a JEOL JSM 4900 LV scanning electron microscope.

### 4.3 Cell culture and infection

The human colonic carcinoma cell line T84 (ATCC CCL‐248) was cultured in DMEM/F‐12 (1:1 mixture, Sigma) supplemented with 10% foetal bovine serum and 2.5 mM L‐glutamine (Sigma). Cells were used between passage 45 and 65. For infections in 24 well plates, T84 cells were seeded at a density of 1.5 × 10^5^ cells/well and grown for 6 to 7 days until full confluency. Cells were infected with 10 μl EAEC overnight culture (~10^7^ CFU) for indicated time periods. After removal of nonadherent bacteria by three washes in sterile PBS, adherence was quantified by lysing cell monolayers in 1% Triton X‐100 in PBS for 10 min and plating serial dilutions on LB agar plates for quantification of CFU. To determine the influence of host cell contact on EAEC gene expression, 3.5 × 10^5^ T84 cells were seeded out in 12 well plates and grown to full confluency (7 days). For infection, 10 μl EAEC overnight culture were either added directly to the cell monolayer or prevented from direct cell contact by insertion of a Transwell insert (12 mm diameter, 0.4 μm pore; Corning Costar). In addition, bacteria were incubated in Transwell plates without T84 cells. All incubations were performed at 37°C in a 5% CO_2_ atmosphere.

### 4.4 Vertical diffusion chamber

Infections in the VDC system were performed as described previously (Tran et al., 2014). Briefly, 5 × 10^5^ T84 cells were seeded on collagen‐coated Snapwell filter inserts (12 mm diameter, 0.4 μm pore; Corning Costar). Transepithelial electrical resistance was monitored using an EndOhm chamber and EVOM resistance meter (WPI), and values of 1,000 to 2,000 Ω × cm^2^ after 11 to 14 days indicated polarisation of the epithelium. After mounting the Snapwell inserts in the VDC apparatus, apical compartments were perfused with anaerobic (5% CO_2_, 5% H_2_, and 90% N_2_) or AE (5% CO_2_ in air) gas mixture whereas basal compartments were maintained under AE conditions. Apical chambers were inoculated with 10 μl EAEC overnight culture and incubated for indicated time periods. For assays without T84 cells, empty Snapwell supports were used to connect both half chambers.

### 4.5 Immunofluorescence staining

After removal of nonadherent bacteria, cells were fixed in ice‐cold ethanol for 15 min and blocked/permeabilised with 0.1% Triton X‐100/0.5% bovine serum albumin in PBS for 20 min. Samples were incubated in polyclonal goat anti‐*E. coli* (1:200, Abcam) for 1 hr, followed by detection in Alexa Flour 568‐conjugated donkey anti‐goat IgG (1:400, Invitrogen) and staining of filamentous actin with fluorescein isothiocyanate‐conjugated phalloidin (1:200, Sigma) for 30 min. Filters were mounted in Vectashield (Vector Laboratories) and examined with an Axio Imager 2 microscope (Zeiss).

### 4.6 RNA isolation and quantitative reverse transcription PCR

RNA from nonadherent bacteria was stabilised in 2% acidic phenol/18% ethanol in water, and RNA from adherent EAEC was enriched by differential lysis of infected cell monolayers in 3% Triton X‐100/2% acidic phenol/18% ethanol in water. After incubation on ice for 30 min, bacteria were pelleted by centrifugation and stored at −70°C until further analysis. RNA was extracted using the RNeasy Mini kit with on‐column DNase digestion (Qiagen). RNA concentration and purity was determined using a NanoDrop ND‐1000 spectrophotometer (Fisher Scientific), and RNA integrity was confirmed by agarose gel electrophoresis. For cDNA synthesis, 1 μg RNA was reverse‐transcribed using qScript cDNA supermix (Quanta BioSciences). Primers specific for EAEC genes were designed using Primer3 and PrimerBLAST software and purchased from Sigma‐Genosys (Table 3). Quantitative PCR was carried out using SYBR Green JumpStart Taq ReadyMix (Sigma) in an ABI7500 Taqman lightcycler (Applied BioSciences) applying the following cycling parameters: 2 min at 95°C, 30 s at 95°C, 30 s at 60°C, 35 s at 72°C (40 cycles), and 5 min at 72°C. Product specificity was confirmed by melt curve analysis and agarose gel electrophoresis. Relative quantification of gene expression was performed using the comparative Ct method. Genes encoding DNA gyrase subunit A (*gyrA*) and glucans biosynthesis protein G (*mdoG*) were selected as housekeeping genes based on evaluation of four E. coli reference genes (Figure S1). Ct values for genes of interest were normalised using the geometric mean Ct of the two reference genes. Fold expression levels in treated samples were calculated relative to matched non‐treated controls using the formula 2^−ΔΔCt^.

**Table 3:** Primer sequences used in this study

Gene	Primer sequence (5′‐3′)	
	Forward	Reverse
Reference		
*gyrA*	CCGAAGTTACCCTGACCGTC	GGTGACTCGGCGGTTTATGA
*mdoG*	AATGCGTTGGTTGAGTGCTG	CCCGGCTAAGGATTGAGCTT
Virulence		
*aggR*	AATTCGGACAACTACAAGCATCT	CAACAGCAAATCCATTTATCGCA
*pic*	AATGCCCTGTCACTTCCCAG	TCGCTGAAAGACGCTGACTT
*hlyE*	GGCTATCTAACGCCAGCAGT	GCATCCGCCCAGAAAGACAT
*aap*	CGGGTCCACATTATCTGCGT	TGGCATCTTGGGTATCAGCC
*aafA*	ACACCGGCTACAAATCGTGA	TTGACCGTGATTGCCTTCCC
*aggA*	GACAATCCGCCTCACCGTTA	AGACCCTTGCACCGCTTTTA
*pet*	TGAACTCGATGGCCTTGACC	CCGGACTCAAACATGGCAGA
*astA*	GACGGCTTTGTAGTCCTTCCA	GAAGGCCCGCATCCAGTTAT
Respiration		
*cyoA*	CCAGACCACAGCTTCCACTT	TTCCCGCAATCTTGATGGCT
*cydB*	ACACTGGTCTGTTTCGCACT	GTGGGTTAGAGGCTGCGTAA

### 4.7 Construction of GFP reporter strains and analysis of promoter activity

The promoter fragments *aafD*100 and *aap*500 were amplified upstream of *aafD* and *aap*, respectively, from EAEC 042 as described previously (Yasir et al., 2018). PCR fragments were cloned into the low copy number GFP reporter plasmid pRW400 via EcoRI and HindIII restriction sites (Alsharif et al., 2015), and constructs were verified by Sanger DNA sequencing. As EAEC 042 is naturally tetracycline‐resistant, plasmids were transformed into a 042 mutant strain lacking *tetA* (DFB042TC). For preparation of electrocompetent bacteria, EAEC DFB042TC was grown in 2X YT broth overnight, subsequently diluted 1:100 in 2X YT supplemented with 700 mM ethylene diamine tetraacetic acid and grown to an OD_600_ of 0.2–0.3. After washing and concentrating cultures in ice‐cold 10% (*v*/v) glycerol, bacteria were electroporated at 2.5 kV using a Gene Pulser II (BioRad), and recombinants were selected on LB agar containing tetracycline. Infections of confluent T84 cells in well plates were performed as described above. Nonadherent bacteria were removed, pelleted by centrifugation, and resuspended in 1% Triton‐X100 in PBS. Cells with adherent EAEC were washed in PBS and lysed in 1% Triton X‐100. GFP expression was quantified by transferring sample aliquots to black 96 well plates (Greiner Bio‐One) and measuring fluorescence at 485 nm excitation/520 nm emission using a FLUOstar Optima Fluorescence Plate Reader (BMG Labtech). Fluorescence was normalised against CFU in respective samples.

### 4.8 SDS‐PAGE and Western Blotting

Lysates of nonadherent EAEC were prepared by suspending bacterial pellets in reducing SDS‐PAGE sample buffer. For adherent EAEC, infected T84 monolayers were lysed in ice‐cold lysis buffer (50 mM Hepes pH 7.4, 50 mM NaCl, 1% Triton X‐100) containing protease inhibitor cocktail (1:200, Sigma). After heat denaturation, proteins were separated in 15% SDS‐polyacrylamide gels and transferred to PVDF membranes (VWR) using a Mini‐PROTEAN Tetra Cell device (Bio‐Rad). Membranes were blocked in 5% skimmed milk in TBS/0.05% Tween‐20 for 1 hr and incubated in polyclonal rabbit anti‐dispersin (1:5,000; kindly provided by Christopher Icke, University of Birmingham) overnight at 4°C followed by HRP‐conjugated goat anti‐rabbit IgG (1:20,000; Sigma) for 30 min. Membranes were developed by enhanced chemiluminescence (Immobilon Western, Millipore) and imaged with a FluorChem E Imager (ProteinSimple). Densitometric analysis of band intensities was performed using ImageJ software (https://imagej.nih.gov/ij/).

### 4.9 Statistics

Statistical analysis was performed using GraphPad Prism software (version 5.04). Student's paired *t*‐test was used to determine differences between two groups. One‐way or two‐way ANOVA with Tukey's multiple comparisons test was used for multiple groups. A *P* value of <0.05 was considered significant.

## Supporting information


**Figure S1:** Evaluation of bacterial housekeeping genes for qPCR. EAEC strains 17‐2 or 042 were incubated in the VDC system for 3 h under aerobic (AE) or microaerobic (MA) conditions. Expression of selected E. coli housekeeping genes in planktonic bacteria was determined by qPCR and is indicated as cycle threshold (C_T_) value (*n* = 5 in duplicate).
