# Supplementary figures and images for: Oxygen and contact with human intestinal epithelium independently stimulate virulence gene expression in enteroaggregative Escherichia coli
Source: Cell Microbiol. 2019 Feb 15;21(6):e13012. doi: 10.1111/cmi.13012 (PMC6563437; doi:10.1111/cmi.13012)

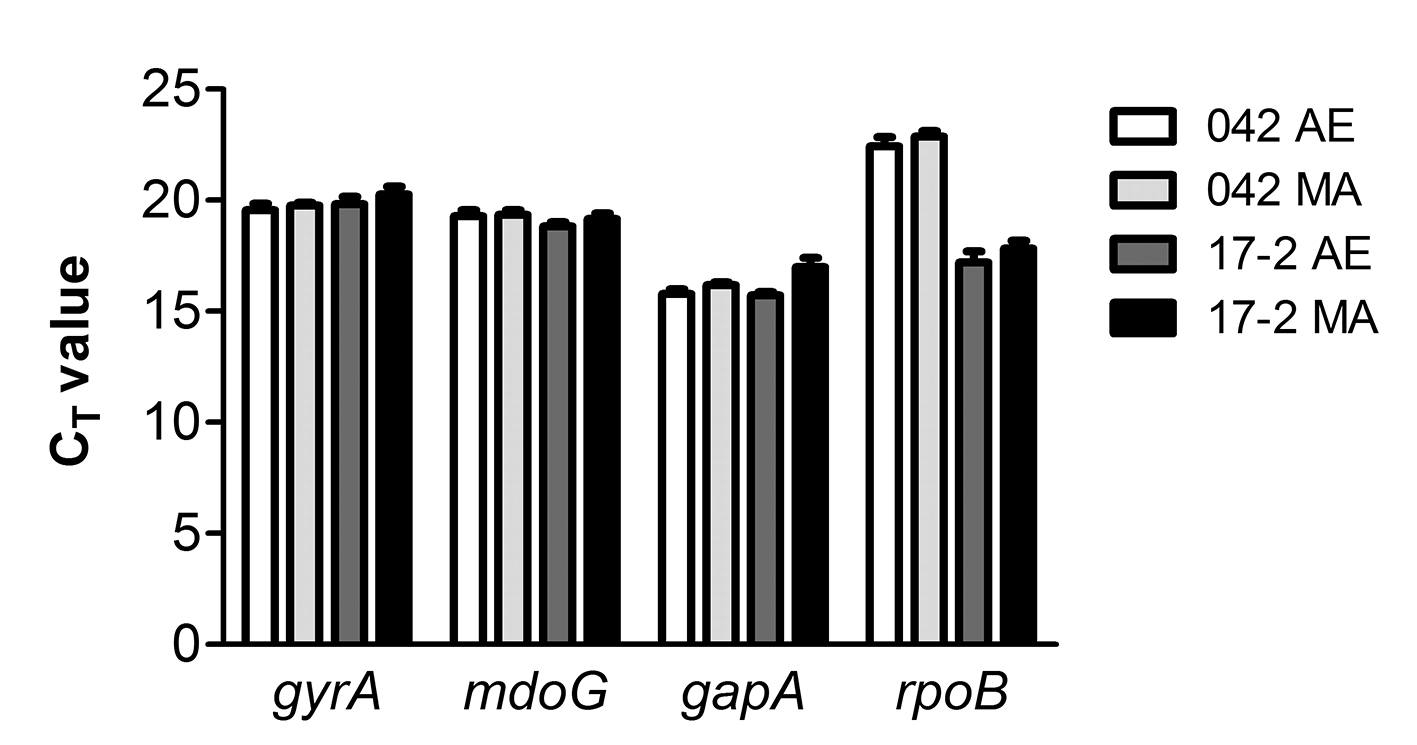

Supplement: Supplementary file 1 — Figure S1: Evaluation of bacterial housekeeping genes for qPCR. EAEC strains 17‐2 or 042 were incubated in the VDC system for 3 h under aerobic (AE) or microaerobic (MA) conditions. Expression of selected E. coli housekeeping genes in planktonic bacteria was determined by qPCR and is indicated as cycle threshold (CT) value (n = 5 in duplicate). [file CMI-21-na-s001.tif]
